# Supplementary material for: Comparison Between Electroporation at Different Voltage Levels and Microinjection to Generate Porcine Embryos with Multiple Xenoantigen Knock-Outs
Source: Int J Mol Sci. 2024 Nov 5;25(22):11894. doi: 10.3390/ijms252211894 (PMC11593736; doi:10.3390/ijms252211894)
Supplement: Supplementary file 1 [file ijms-25-11894-s001.zip › ijms-3277309-supplementary.pdf]

Supplementary Material

Table S1. sgRNAs considered for gene editing

Gene Name	sgRNA Name	sgRNA Sequence	Exon #
GGTA1	GGTA1-41	5´-GATGCGCATGAAGACCATCG-3´	8
CMAH	CMAH 10-3	5´-CTACAAGGCTCGGCTGGTGA-3´	10
	CMAH 10-4	5´-GAGGAATGGAAAGCCCAATT-3´	10
B4GALNT2	B4GalNT2Ex3-Egen	5´-TCCTCAGGTTCACTGCGGGG-3´	3
	B4GALNT2EX3#1	5´- GATGCCCGAAGGCGTCACAT-3'	3

Table S2. Parameters utilized for embryo electroporation

Parameters											
Poring Pulse						Transfer Pulse					
V	Length (ms)	Interval (ms)	No.	D.Rate (%)	Polarity	V	Length (ms)	Interval (ms)	No.	D.Rate (%)	Polarity
30/35/40 (variable)	3.5	50	4	10	+	5	50	50	5	40	+/-

V: Voltage in volts, D: Decay

Table S3. Primer characteristics for xenoantigen and transgene amplification

Organism	Gene	Direction	Sequence (5' - 3')	Annealing Temperature (°C)	Size (bp)
Human	A20	Forward	TGGGACTCCAGAAAACAAGG	58	950/231
		Reverse	GTCCTTTTGGCCTCATG		
Pig	GGTA1	Forward	CTAGAAATCCCAGAGGTTAC	59	497
		Reverse	GGGTGTTCCCCCAAAAATGG		
	CMAH	Forward	AGCCACTAATGACAAGGAGCT	58	288
		Reverse	GCCATACTTGTCTGCTGGGT		
	B4GALNT2	Forward	ACTCTGCATGCCAAGAGTTAAGA	62	491
		Reverse	CCTGGAGACTTTGAGAGCCG		

Table S4. Sequence reads considered for mutational analysis

	E30V	E35V	Microinjection	NA
GGTA1	19	19	20	2
CMAH	20	19	20	1
B4GALNT2	19	19	18	4
Total	58	57	58	7

Note: NA represents the number of no available reads for analysis

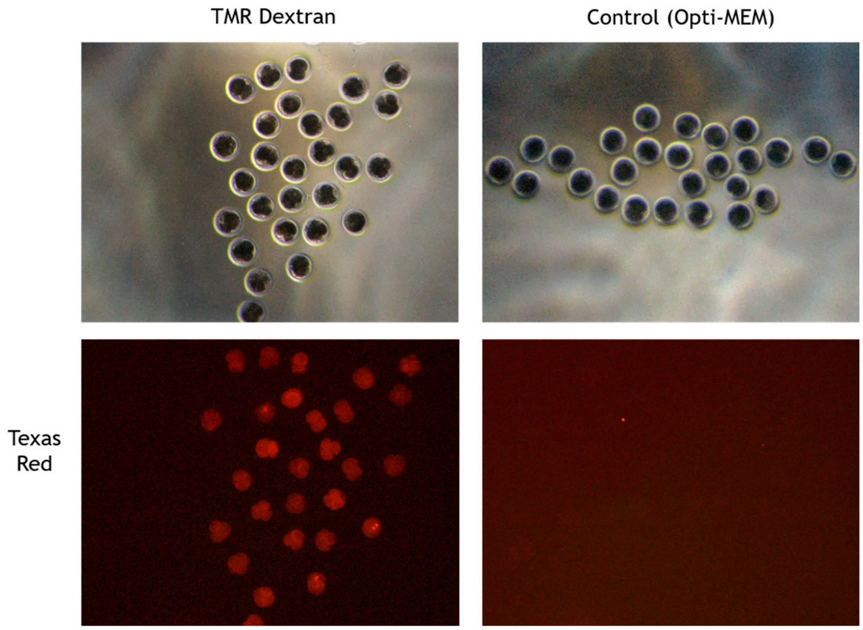

Figure S1. Fluorescence recordings of TMR-D electroporation at 30 volts of parthenogenetically activated zygotes 1 day after electroporation (10x magnification).

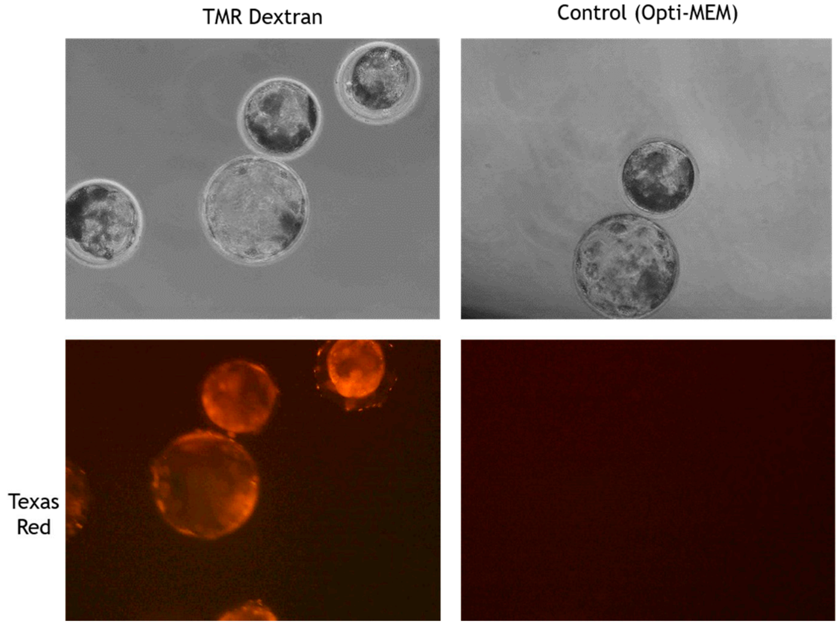

Figure S2. Fluorescence recordings of TMR-D electroporation at 30 volts in IVF-derived embryos, 5 days post-electroporation (10x magnification).

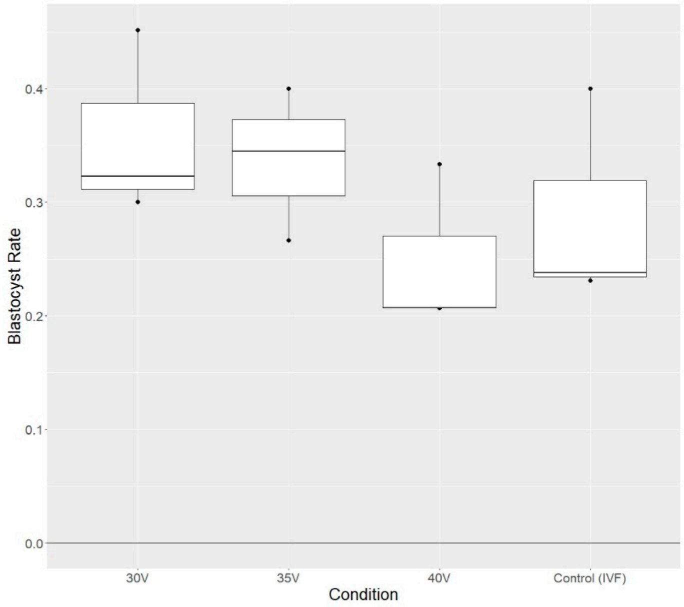

Figure S3. Analysis of BR in IVF-derived zygotes at 30, 35 and 40 volts for TMR-Dextran delivery

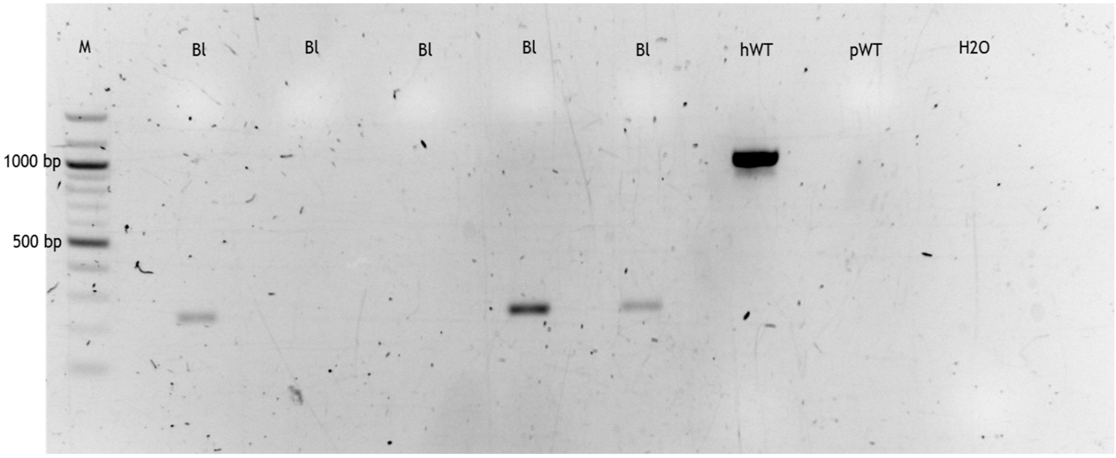

Figure S4. The human A20 transgene (231 bp) is found in three out of five RNP-targeted blastocysts from a boar carrying the expression cassette containing human CD46, CD55, CD59, A20, and HO-. M: Marker, Bl: Blastocyst, hWT: human wild-type pluripotent cells (950bp), pWT: wild-type porcine fibroblast cells. Original Figure (Suppl. Figure 14)

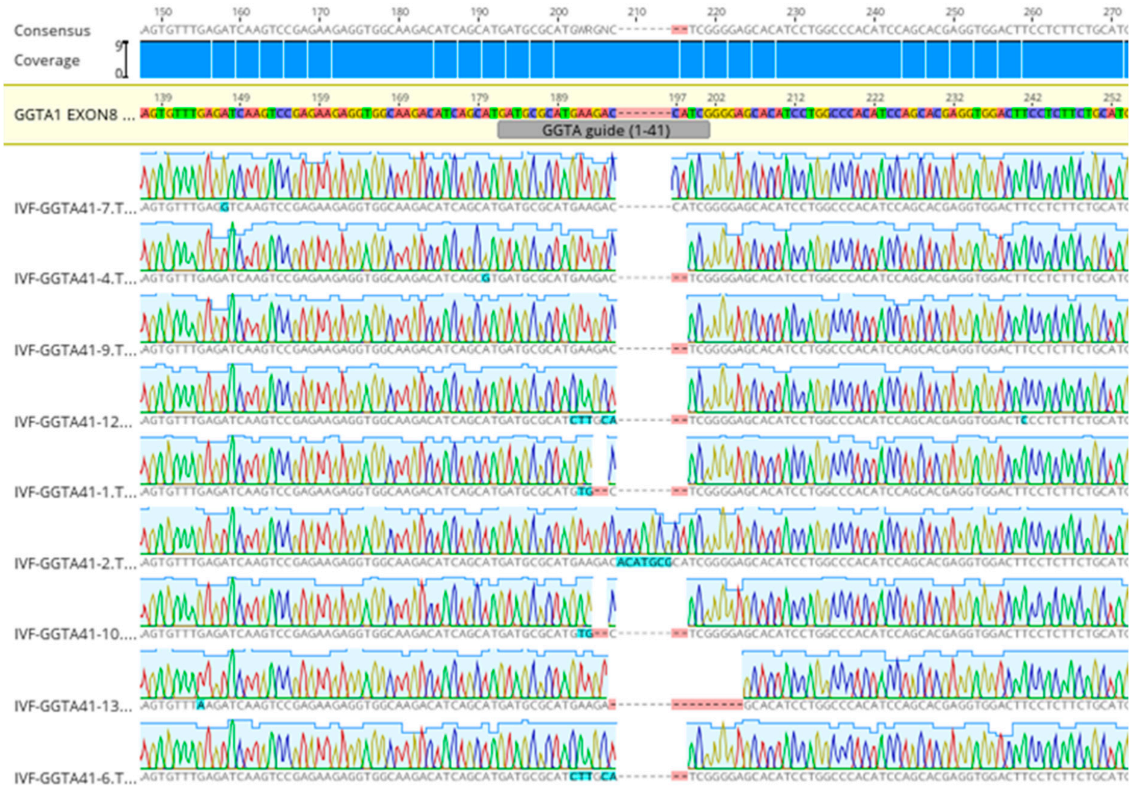

Figure S5. Allele specific sequencing of GGTA1 in IVF-derived blastocysts electroporated with sgRNA GGTA1 1-41.

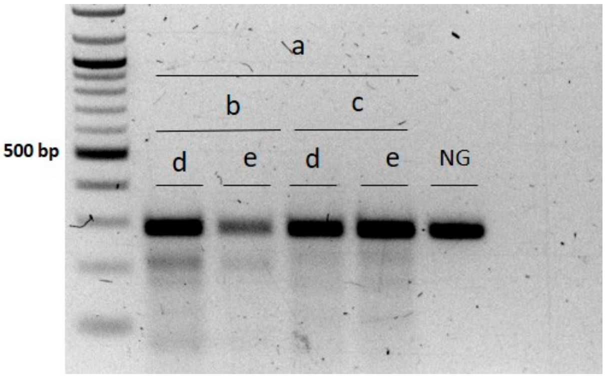

Figure S6. Editing of CMAH for diverse RNP combinations.

a-d Respective sgRNAs utilized in RNP delivery.

a GGTA1 1-41

b CMAH10-3

c CMAH 10-4

d Bgalnt2EX3#1

e B4galnt2Ex3-Egen

NG= No sgRNA delivered

Original Figure (Suppl. Figure 15, 16)

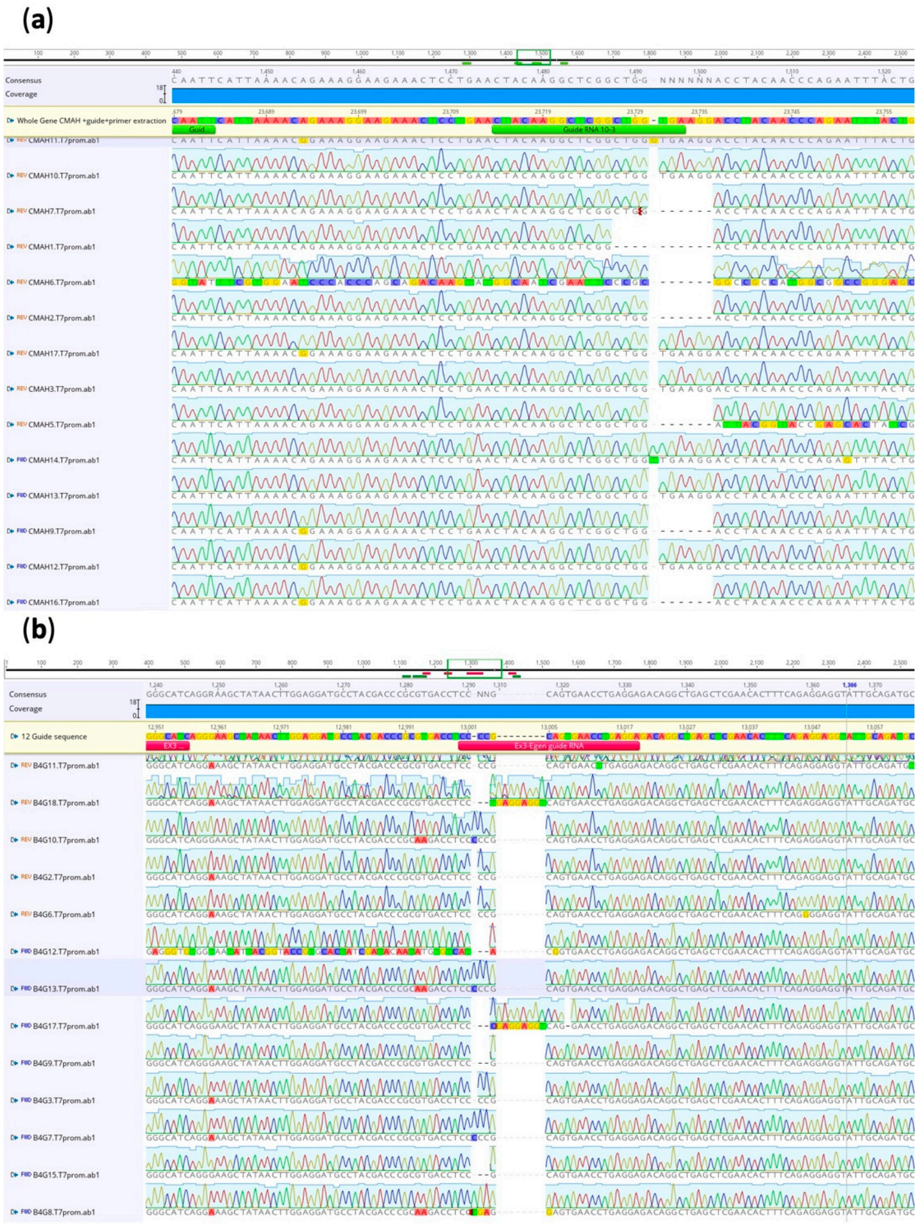

Figure S7. Allele specific sequencing of CMAH (**a**) and B4GALNT2 (**b**) in IVF-derived blastocysts electroporated with RNP complex combination: abe.

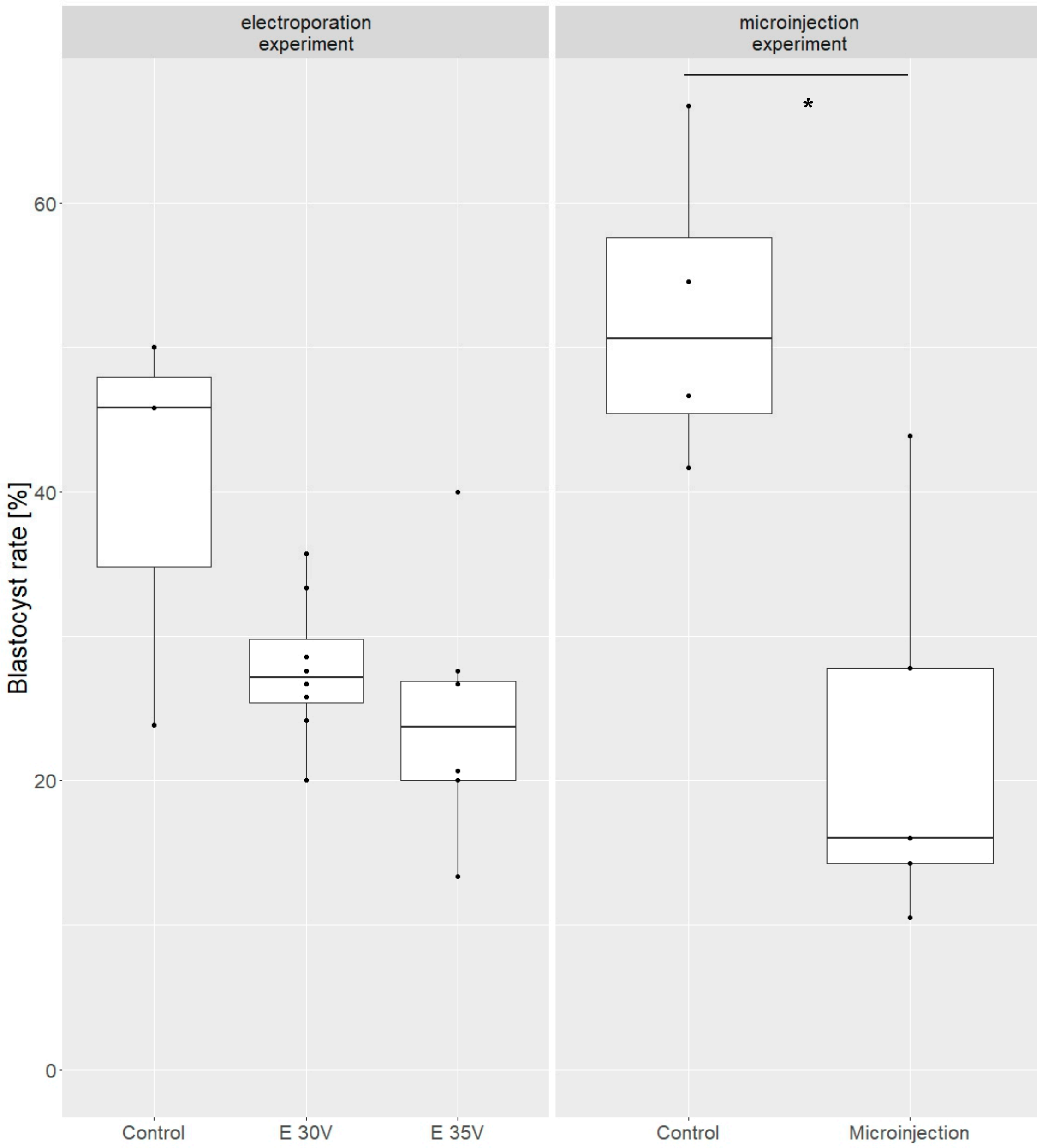

Figure S8. Comparison of mean BR across RNP delivery groups

The average BRs are compared between the treatment groups (gene-edited vs. Control) for the three delivery methods. A significant difference determined by t-test is observed in the microinjection group compared to its control counterparts ($p < 0.01$). BRs from E30V and E35V sharing the same control reference, do not display any significant differences when compared to this control or between each other.

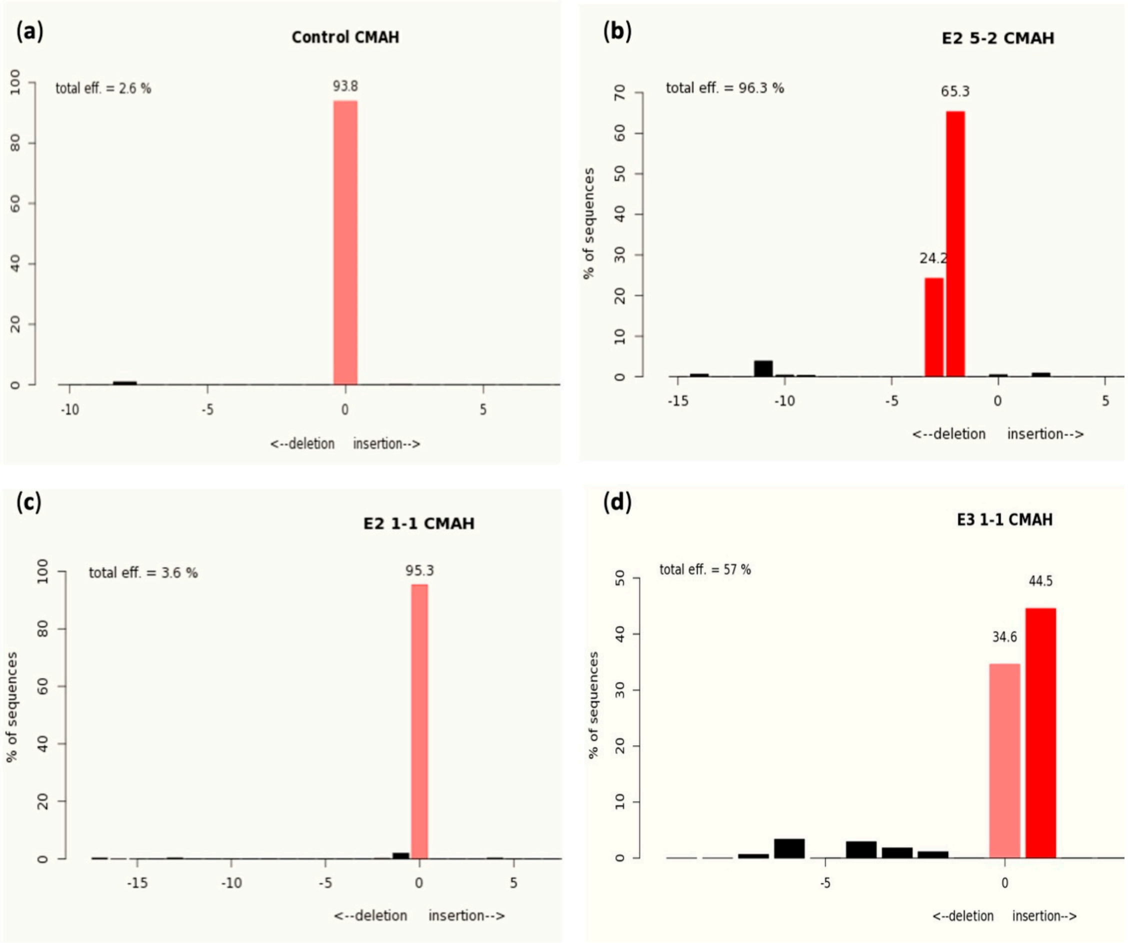

Figure S9. Genetic variant classification methodology

Illustration of the genetic variant classification methodology utilizing TIDE output, exemplified by the xenoantigen CMAH. (**a**) Mutation analysis of a fluorescent IVF blastocyst control without RNP complex delivery. The bright red column represents the WT allele frequency, originating from the 0 value on the X-axis. (**b**) Biallelic disruption is recognized when the targeted mutation is present (bright red columns differing from 0), and the wild-type allele is absent in the analysed sequence. Conversely, unsuccessful editing (**c**) is depicted by the presence of the WT genotype with no traces of the targeted mutation. Mosaicism (**d**) is characterized by a spectrum of indels, showcasing a combination of mutation and WT alleles.

Note: Editing efficiency is represented by total eff. in each figure.

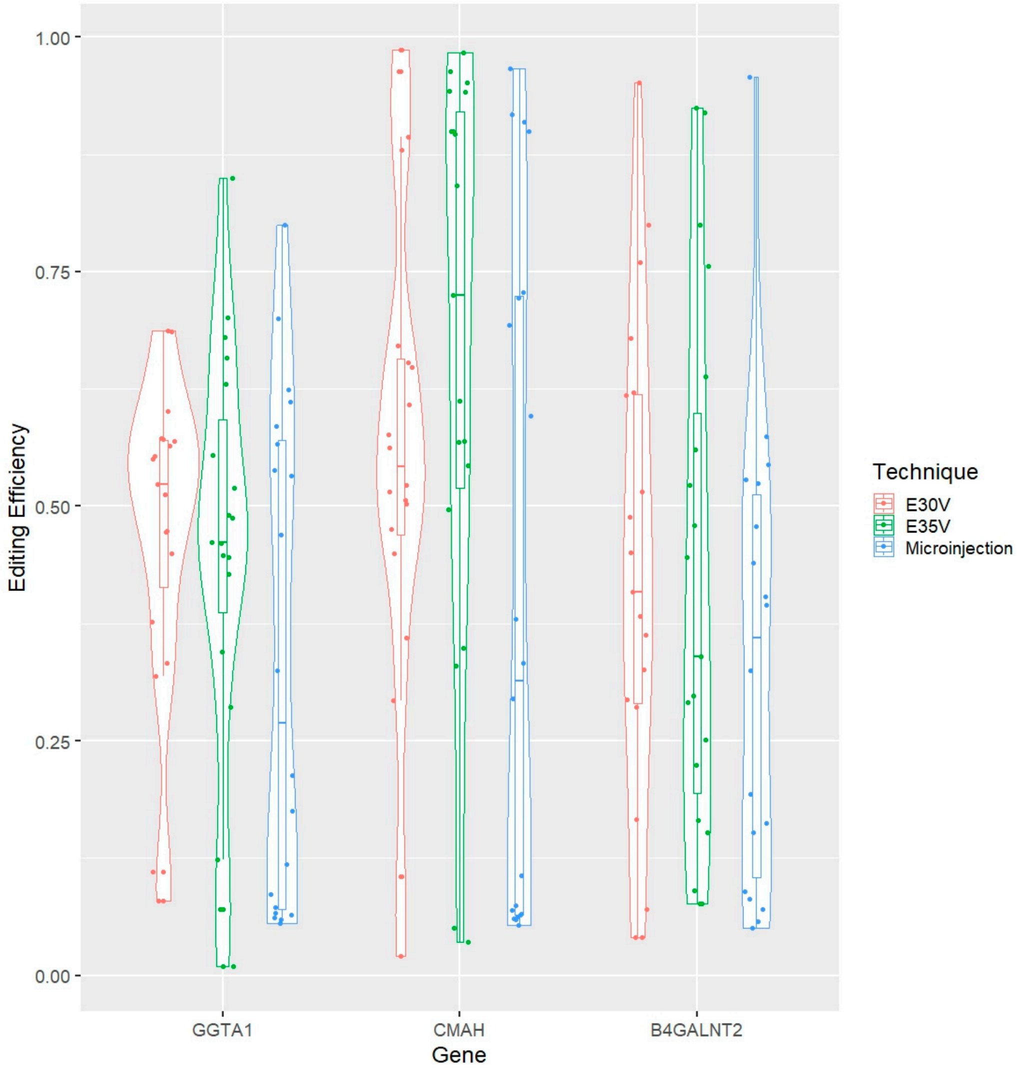

Figure S10. Xenoantigen-specific Indel Scores across delivery techniques in analyzed blastocysts
Comparison of Indel Score values for each targeted gene locus among delivery techniques determines non-uniform variations in specific genes. No significant differences were found between techniques for individual genes (Wilcoxon test).

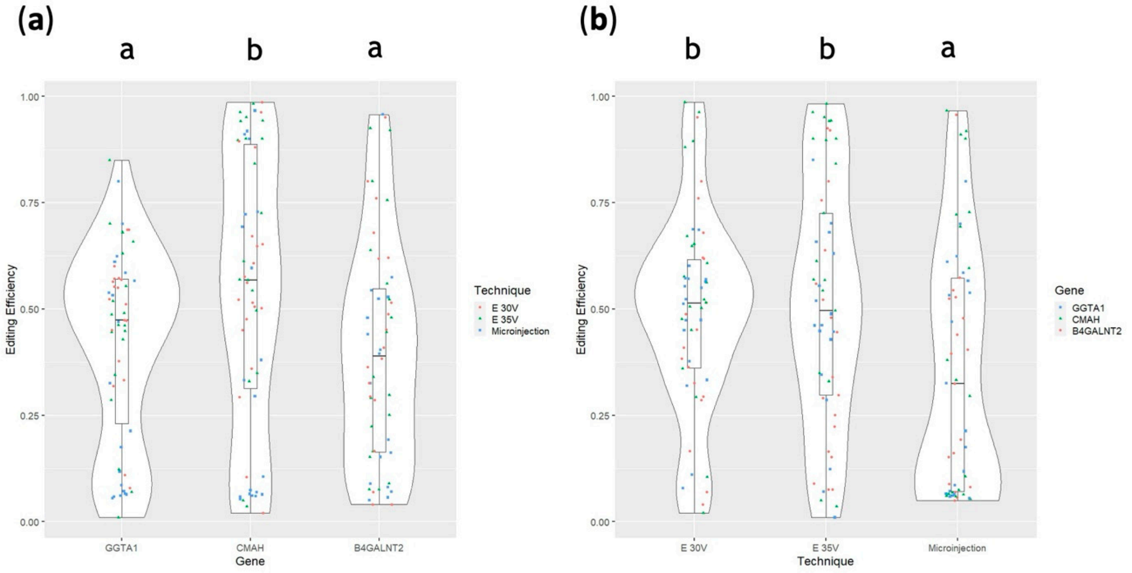

Figure S11. The overall indel score values are compared across gene loci (**a**) and techniques (**b**). Values in the same subfigure with different superscripts are significant (Wilcoxon test).

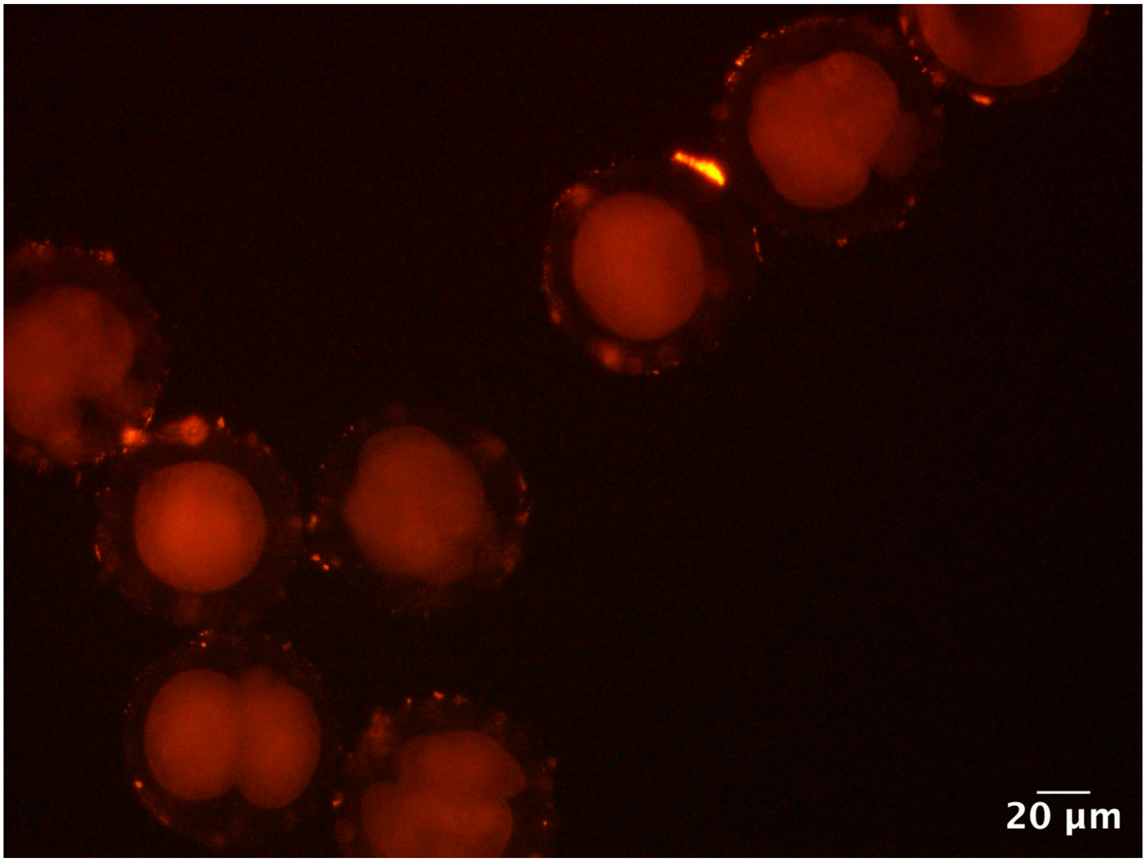

Figure S12a. Microscopical recording of zygotes 24 hours after TMR-D delivery at 30 V

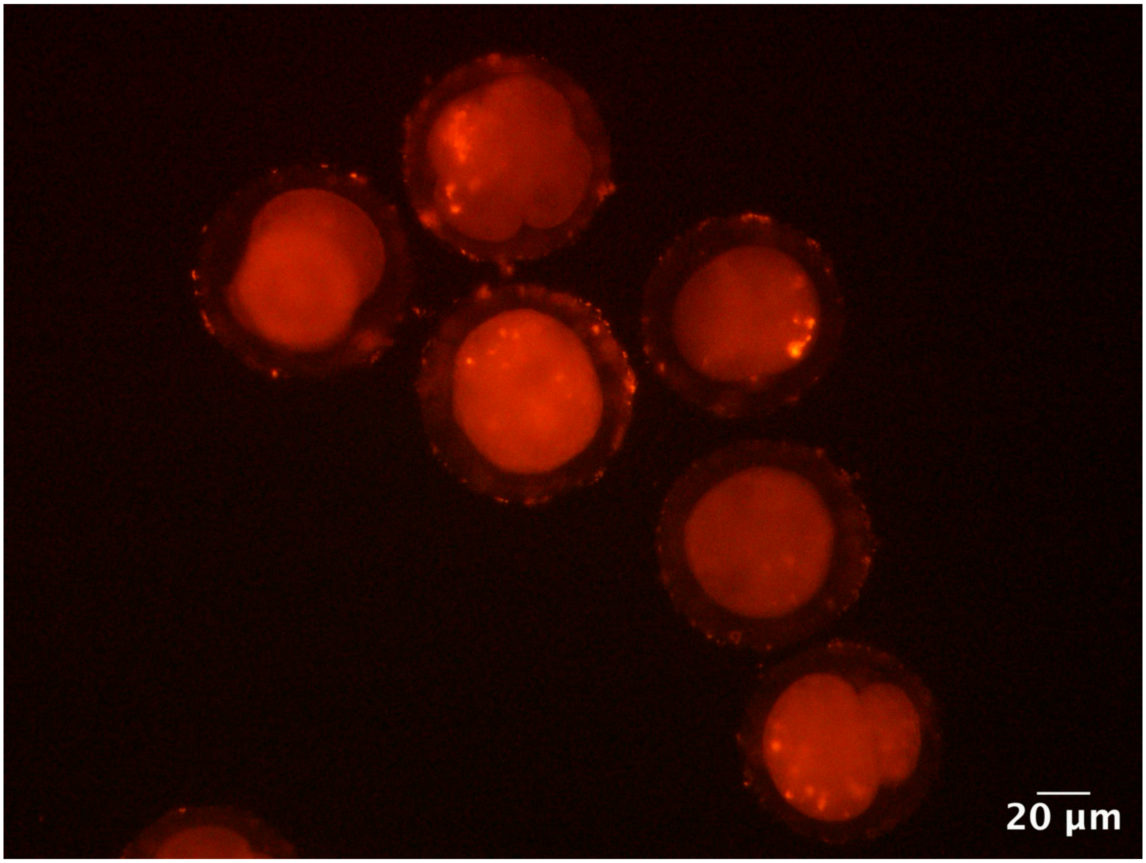

Figure S12b. Microscopical recording of zygotes 24 hours after TMR-D delivery at 35 V

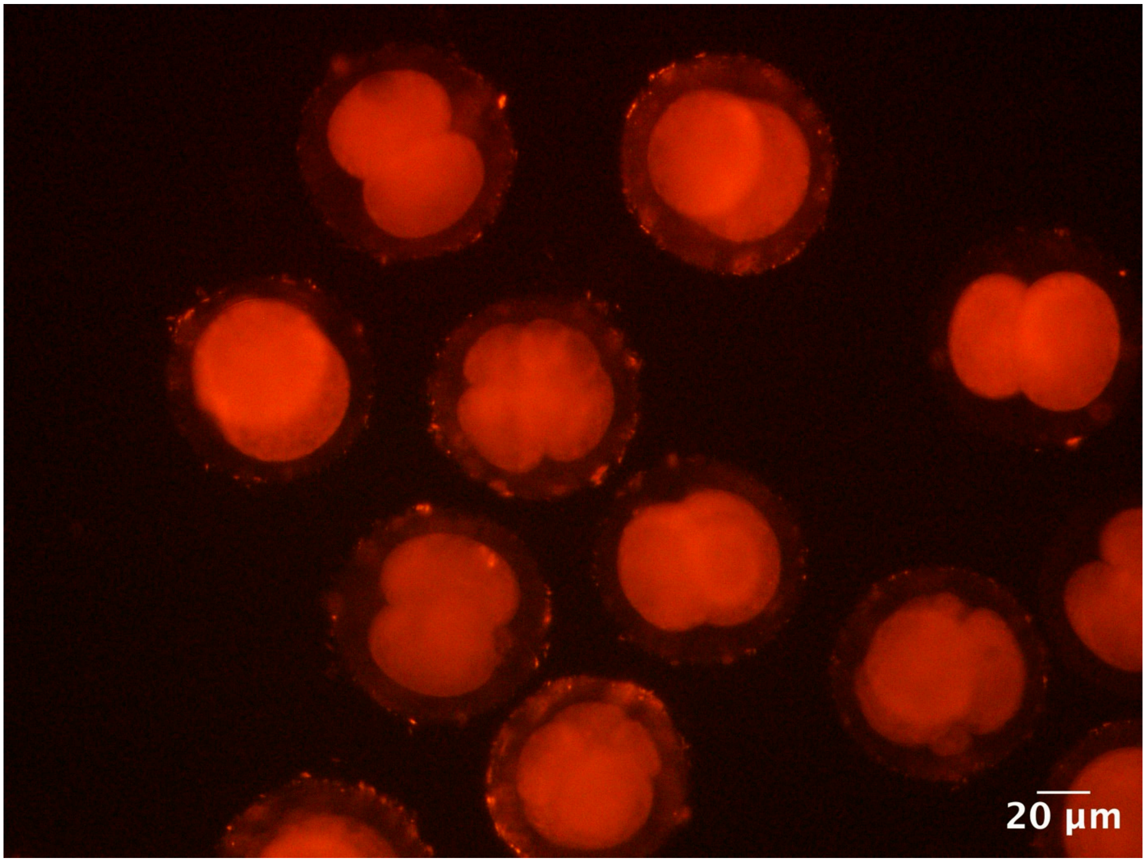

Figure S12c. Microscopical recording of zygotes 24 hours after TMR-D delivery at 40 V

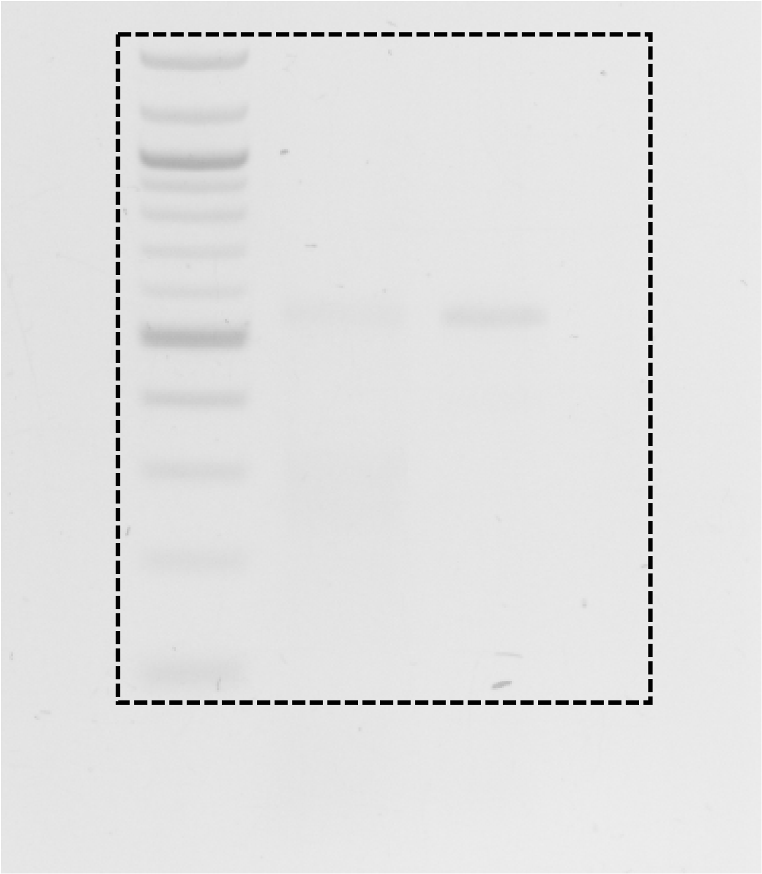

Figure S13 a. Original image of Figure 2a; T7E1-digested fragment confirms CRISPR/Cas9-induced mutations in blastocysts resulting from GGTA1 RNP delivery via electroporation.

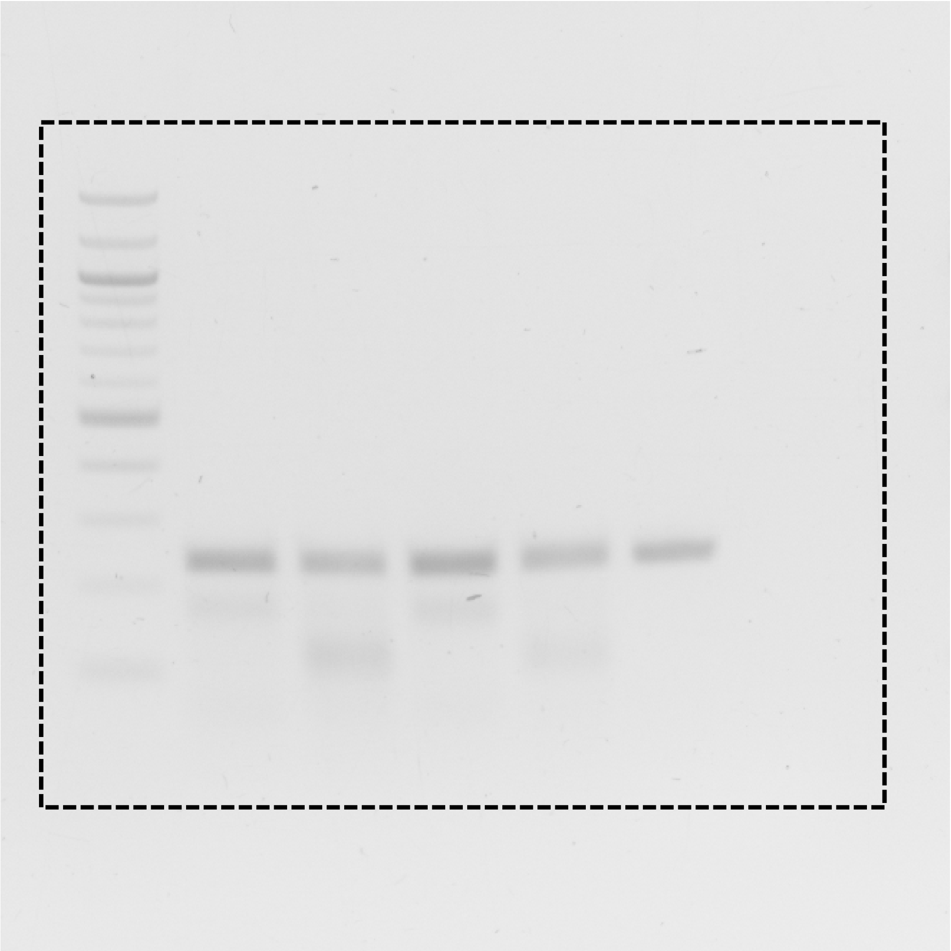

Figure S13b Original image of Figure 2b. Editing of B4GALNT2 for diverse RNP combinations (abd, abe, acd, ace).

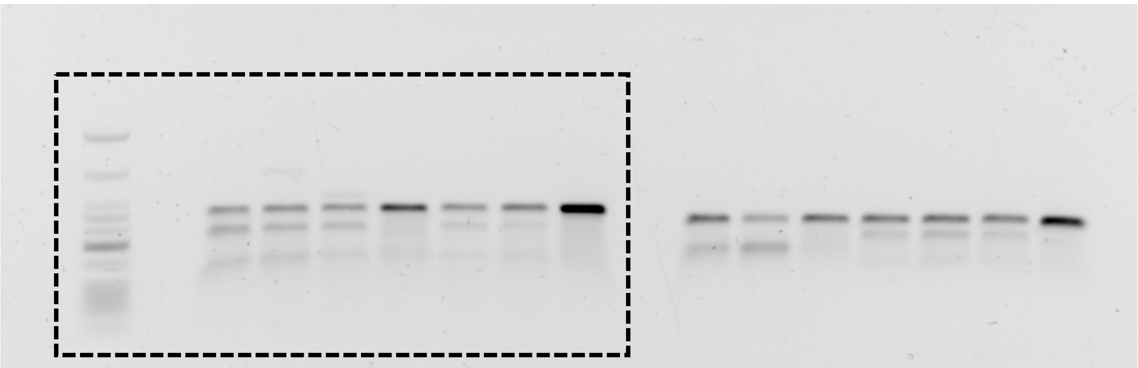

Figure S13c. Original image of Figure 2c. The digestion of CMAH, conducted in triplicate experiments, reveals a stronger digestion band in the presence of B4GALNT2Ex3-Egen (abe) within the RNP compared to BGALNT2EX3#1 (abd)
